# Assessment of YouTube Videos in Patient Education for Coronary Artery Disease: A DISCERN-Based Cross-Sectional Analysis

**DOI:** 10.7759/cureus.62986

**Published:** 2024-06-23

**Authors:** Jasneel S Kahlam, Alexander Sacher, Chanpreet Singh, David F Lo, John P Reilly

**Affiliations:** 1 Internal Medicine, Stony Brook Southampton, Hackettstown, USA; 2 Internal Medicine, New York-Presbyterian, Queens, USA; 3 Basic Medical Science, New York Institute of Technology College of Osteopathic Medicine, Old Westbury, USA; 4 Basic Sciences, American Preventative Screening and Education Association, Stratford, USA; 5 Cardiology, Stony Brook University, Stony Brook, USA

**Keywords:** patient education, youtube, cabg, cardiac catheterization, coronary artery disease treatment, coronary artery disease

## Abstract

Introduction

Cardiovascular disease has one of the highest mortality rates and continues to grow. Therefore, it is important for the medical community to get involved in widespread patient education efforts. As technology has steadily advanced, YouTube (Google LLC, Mountain View, California, United States) has become a popular source for patients to gather medical information. In this study, we aim to assess the quality of YouTube videos pertaining to coronary artery disease.

Methods

We searched the following key terms on June 20, 2023, using the view count filter: coronary artery disease, coronary artery disease treatment, cardiac catheterization, and coronary artery bypass grafting (CABG). The top twenty videos for each keyword were recorded. After videos that were over 20 minutes, non-English, procedural videos without words, and duplicates were excluded, forty-five videos remained. Each video was assessed by three viewers using the DISCERN criteria (http://www.discern.org.uk). Numerical data was averaged into composite scores. Two-sided t-tests and one-way analysis of variance (ANOVA) tests were used to compare mean ratings between groups. A Spearman correlation was done to compare each of the following terms to one another: overall quality of videos, total likes a video received, and total views.

Results

The mean ratings for coronary artery disease, coronary artery disease treatment, cardiac catheterization, and CABG were 2.30, 2.60, 2.05, and 2.92, respectively, with an overall mean of 2.42. The means between coronary artery disease and coronary artery disease treatment were significantly different (p adj = 0.01). The overall rating for videos with board-certified physicians was significantly higher than those without a board-certified physician (p < 0.001). There was a low correlation between likes and overall ratings (0.03) and views and overall ratings (-0.068).

Conclusion

The videos on coronary artery disease, coronary artery disease treatment, cardiac catheterization, and CABG had poor overall quality based on DISCERN criteria. The overall ratings from videos with board physicians are higher than those from non-physicians, suggesting that physicians should be encouraged to create content about important medical conditions. There was also a low correlation between the overall quality of a video and the likes and views, respectively, indicating a disconnect between what the public values and the actual value of a video.

## Introduction

With an escalating mortality rate, coronary artery disease (CAD) remains a critical global health challenge. Annually, CAD contributes to 610,000 deaths and costs our healthcare system 200 billion dollars [1]. However, with the right treatment and lifestyle modifications, the risks of developing this disease can be significantly decreased. Treatment of this disease varies in severity, from lifestyle modifications to interventions such as cardiac catheterization and surgery. To decrease the need for invasive procedures, early patient education is crucial in preventing the progression of disease. Patient education has been shown to promote positive changes in exercise, healthy diets, and smoking cessation [2]. If the patient fails to optimize diet and exercise, the next step would be medication. If symptoms persist after medication, the patient may be a candidate for a cardiac catheterization, which has been shown to improve symptoms and quality of life [3,4]. If the cardiac catheterization fails to revascularize the affected region of the heart or if the patient has multiple vessel disease, the patient could be a candidate for coronary artery bypass grafting (CABG). The data has shown that the CABG has improved survival and mortality when compared to percutaneous coronary intervention (PCI) [5,6]. It is the goal of cardiologists and primary care physicians to educate their patients about these interventions.

There are different forms of education, varying from discussions in the physician's office to the use of auditory or visual aids. In one meta-analysis, it was shown that the use of aids such as audiotape recordings or videos was shown to have more of a positive effect in quelling patient worries and increasing overall satisfaction [7]. Additionally, visual decision aids have been effective in acute mechanical thrombectomy in ischemic stroke patients, allowing for more efficient decision-making and overall better stroke outcomes [8,9].

One of the leaders in providing education to patients is YouTube (Google LLC, Mountain View, California, United States), one of the most popular media websites. This has the potential to provide simple access to patient education, especially in underserved communities. However, although YouTube provides free and extensive access to free medical information, this does not guarantee the videos are of high quality. In studies assessing videos for colorectal cancer, fibromyalgia, and pulmonary rehabilitation for COVID patients, it has been shown that although the information may be correct, these videos were seen as having poor quality for patient guidance [10-12]. Additionally, the videos assessed in some studies have been shown to spread misinformation about certain pathologies [13,14]. Even in the field of cardiology, the assessment of YouTube videos for atrial fibrillation and aortic stenosis showed poor quality [15,16]. We hope to assess this trend for YouTube videos on coronary artery disease.

To date, there have only been five studies that have overlapped with this one [17-21]. The study that overlapped the most with this study was Cetin et al. (2023). Although three of the four search terms that they used were similar to this study, they did not evaluate the key terms for each category. Additionally, there was only one viewer, which added more bias to the study [17]. There was one study that investigated the quality of videos regarding CABG, which showed poor quality [18]. However, this video only assessed CABG, not CAD, and the videos were assessed three years prior to this study [18]. There was another study regarding angioplasty and angiography [19]. Although angiography videos are assessed, this study fails to compare scores to those of “coronary artery disease” and is not inclusive of coronary angiography. There was a fourth study that analyzed the quality of YouTube videos for CAD during COVID-19 [20]. Although this analysis included a search term for CAD as well as a comparison between view count and DISCERN ranking, it focused more on COVID-19, assessed the overall quality of all the videos, and only included videos in Turkish [20]. Finally, one study was done to analyze YouTube videos for heart attacks. Although CAD is a major cause of cardiac arrest, it is not the only cause, so it cannot be assumed that all of these videos talk about CAD treatment and procedures [21]. 

The purpose of this study is to compare the overall rating between the search terms mentioned below, assess physician vs. non-physician ratings, and find out if there is a correlation between overall views/likes and the quality of the videos.

## Materials and methods

The search terms "coronary artery disease,", “coronary artery disease treatment,” “cardiac catheterization,” and “CABG” (coronary artery bypass grafting) were searched on June 20th, 2023, using the view counter filter on YouTube. The view counter filter was used instead of the relevance filter because this study wanted to assess the most popular videos that viewers were watching. For each search term, the top 20 videos were included in this study. From the 80 total videos, these videos were excluded if they were over 20 minutes, non-English, or procedural videos. Duplicate videos were assessed and then excluded. 

The remaining videos were then assessed using DISCERN criteria (http://www.discern.org.uk), a validated survey created by Oxford University and the British Library that is used to assess the quality of written consumer health information. The survey included 15 questions assessing the quality of YouTube videos on a scale of one to five, five being the highest quality. The total score is calculated by adding the scores from each question, where a higher DISCERN score indicates better reliability. The Global Quality Survey was not included because it was similar to the scale that we used to quantify the questions from DISCERN. Modified DISCERN was not used because, while it included many of the same questions as DISCERN, it left out some important parameters such as quality of life, benefits, and risks. These categories were important to include because they allowed for a holistic analysis of the video. 

Additionally, the viewers were asked about the length, number of views, likes, dislikes, subscribers, and presence of board-certified physicians. Each of these questions was then assessed to see if they had an impact on the quality ratings found for each video. 

There were three viewers for this study. Of the three viewers, two were internal medicine residents who have spent time on a cardiology rotation, either as a medical student or resident. The third viewer was a third-year medical student who had completed his preclinical education in cardiology. Each viewer was blinded by the other’s answers to prevent any bias. Each of the ratings for each question was averaged and statistically analyzed with two-way t-tests and one-way analysis of variance (ANOVA) tests. Correlation coefficients (r) were calculated using the Spearman test to determine the relationship between likes, views, and overall quality. All statistical analyses were done using IBM SPSS Statistics for Windows, Version 24 (released 2016; IBM Corp., Armonk, New York, United States).

## Results

**Figure 1:**
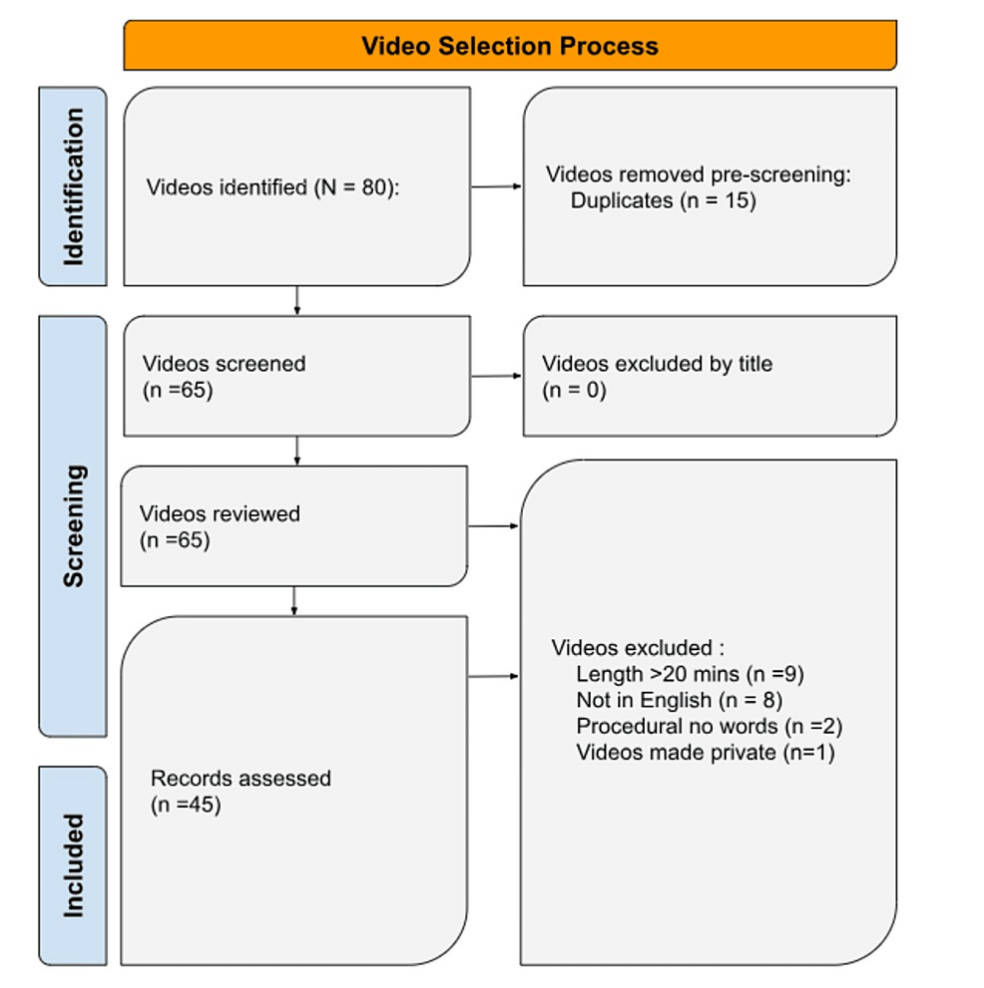
A PRISMA flowchart illustrates the systematic review process for assessing the quality of YouTube videos pertaining to coronary artery disease. The flowchart outlines the selection and screening of studies, including search strategies, eligibility criteria, and the final inclusion of relevant studies for data synthesis and analysis. PRISMA: Preferred Reporting Items for Systematic Reviews and Meta-Analyses

From the initial searches, there were 80 videos included in this video. From these 80 videos, 15 were repeats, nine videos were eliminated for being over 20 minutes, eight videos were non-English, and two videos were procedural, which left 46 videos. During the time of the assessment, one of the videos was made private, so not all of the viewers were able to view the video. This video was eliminated from the study, leaving 45 videos. This is further illustrated in Figure 1. Table 1 shows the average and range of each video's duration, likes, dislikes, and total views.

**Table 1:** Assesses the mean, standard deviation, minimum, and maximum values of each of the following video characteristics: total number of views, likes, dislikes, and duration in seconds.

Video characteristics	Mean ± SD/n (%)	Min/Max
Total number of views	3,181,888±51905	678/41,711,280
Likes	84768±3391	0/3803626
Dislikes	0	0-0
Duration (second)	457.6±2.28	60/1196

Average overall rating for search term and DISCERN question

The overall ratings for each search term and category are included in Table 2. The overall average for the search terms “CABG”, “cardiac catheterization”, “coronary artery disease” and “coronary artery disease treatment” was 2.30, 2.60, 2.05, and 2.92, respectively. Overall ratings for each of the questions are shown in Table 3, and the average rating for each of the videos was 2.42.

**Table 2:** Overall mean for each search category and question. CABG: coronary artery bypass grafting; CAD: coronary artery disease

Question asked in survey	CABG	Cardiac catheterization	Coronary artery Disease	Coronary artery disease treatment
Does the video have clear aims?	3.73	3.48	3.57	3.83
Does the video achieve its aim?	3.97	4.05	3.88	3.96
Is the video relevant? Does it address all the questions that someone would ask? Are recommendations and suggestions appropriate?	3.7	3.5	3.07	3.42
Is it clear what sources of information were used to compile the video?	2.07	2.15	1.93	2.5
Is it clear when the information reported was produced?	1.4	1.33	1.71	1.79
Is there any bias in this video?	3.83	3.98	3.95	4.08
Does it provide additional source of information?	1.37	1.4	2.02	2
Does it refer to any areas of uncertainty?	2.03	2	1.64	2.46
Does it explain how treatment works?	4.73	3.95	2.81	3.88
Does it describe the benefits of treatment?	2.93	2.78	1.98	2.96
Does it describe the risks of treatments?	1.53	2.53	1.12	1.63
Does this video describe what happens when no treatment is used?	1.5	1.5	1.98	2.08
Does it describe how treatment choices could effect overall quality of life?	2	1.98	1.24	1.71
Is it clear that there is more than one option to treat/manage CAD?	2.3	1.58	2.21	2.88
Does the video mention any shared decision making?	1.87	2.25	1.38	2.33
Rate the overall quality of the video on one to five	2.3	2.6	2.05	2.92

**Table 3:** Mean, median, and standard deviation for score of each question. CAD: coronary artery disease

Questions from survey	Mean± standard deviation	Median
Does the video have clear aims?	3.62±1.3	4
Does the video achieve its aim?	3.96±1.12	4
Is the video relevant? Does it address all the questions that someone would ask? Are recommendations and suggestions appropriate?	3.4±1.21	3
Is it clear what sources of information were used to compile the video?	2.12±1.16	2
Is it clear when the information reported was produced?	1.54±1.06	1
Is there any bias in this video?	3.96±1.2	5
Does it provide additional source of information?	1.69±1.28	1
Does it refer to any areas of uncertainty?	1.98±1.14	2
Does it explain how treatment works?	3.76±1.56	5
Does it describe the benefits of treatment?	2.6±1.66	2
Does it describe the risks of treatments?	1.71±1.28	1
Does this video describe what happens when no treatment is used?	1.75±1.07	1
Does it describe how treatment choices could effect overall quality of life?	1.71±1.12	1
Is it clear that there is more than one option to treat/manage CAD?	2.16±1.67	1
Does the video mention any shared decision making?	1.91±1.34	1
Rate the overall quality of the video on one to five	2.42±1.15	2

Comparison of overall rating between categories of videos

The differences in means were compared in Table 4 using a Tukey multiple comparison. There was a significant difference between the means found for coronary artery disease treatment and coronary artery disease (adjusted p = 0.01). 

**Table 4:** Tukey multiple comparisons between each of the search categories. CABG: coronary artery bypass grafting; diff: difference in the mean of DISCERN scores between each categories; Lower: lower range of 95% confidence interval; Upper: upper range of 95% confidence interval

Keywords	Diff	Lower	Upper	Adjusted p-value
Cardiac catherization-CABG	0.3	-0.4	1	0.68
Coronary artery disease-CABG	-0.25	-0.94	0.44	0.78
Coronary artery disease treatment-CABG	0.62	-0.18	1.41	0.19
Coronary artery disease-cardiac catherization	-0.55	-1.19	0.09	0.12
Coronary artery disease treatment-cardiac catherization	0.32	-0.43	1.06	0.69
Coronary artery disease treatment-coronary artery disease	0.87	0.13	1.61	0.01

Overall rating between physicians and non-physicians

**Figure 2:**
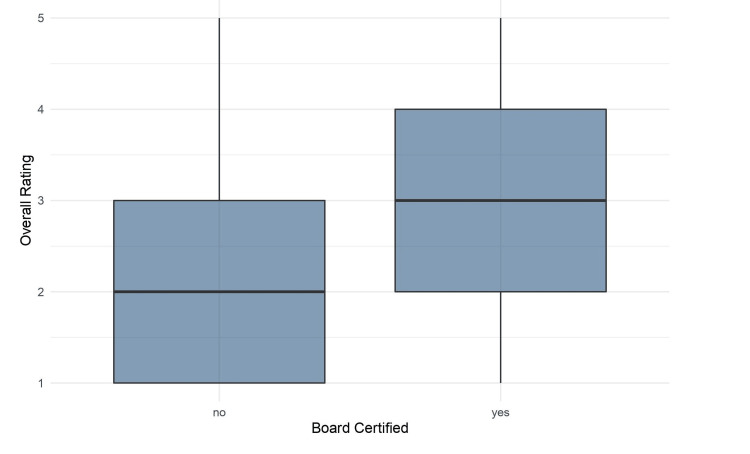
Box-plots of scores for videos with board-certified physicians and videos without board-certified physicians.

A box plot comparing the overall rating between videos with a board-certified physician vs. videos without a board-certified physician is shown in Figure 2. The videos with board-certified physicians showed a significantly higher rating than those without a board-certified physician (3 vs. 2, p-value of <0.0000001).

Overall rating between cardiologist vs. non-cardiologist

**Figure 3:**
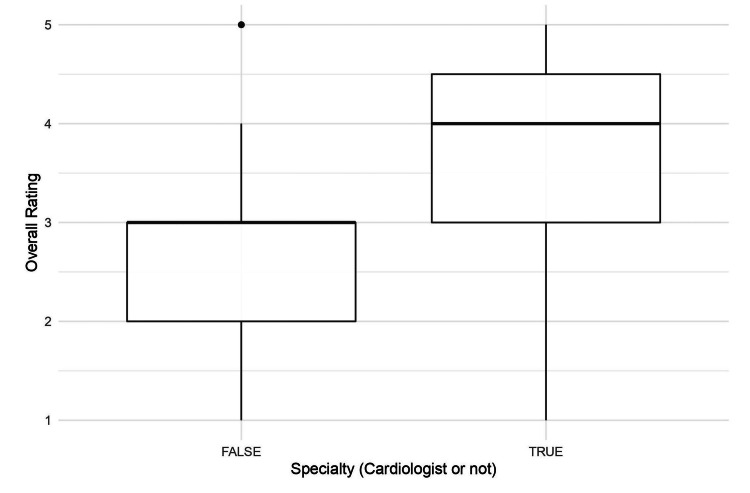
A box plot shows the difference in average rating between videos with cardiologists (interventional cardiologists and cardiothoracic surgeons were included) and videos with physicians who were not cardiologists.

A box plot comparing the average overall rating between cardiologists and non-cardiologist physicians is shown in Figure 3. This plot showed no significant difference between the videos featuring a cardiologist and videos that did not feature a cardiologist (p = 0.3852).

Correlation between overall quality, likes, and views

**Figure 4:**
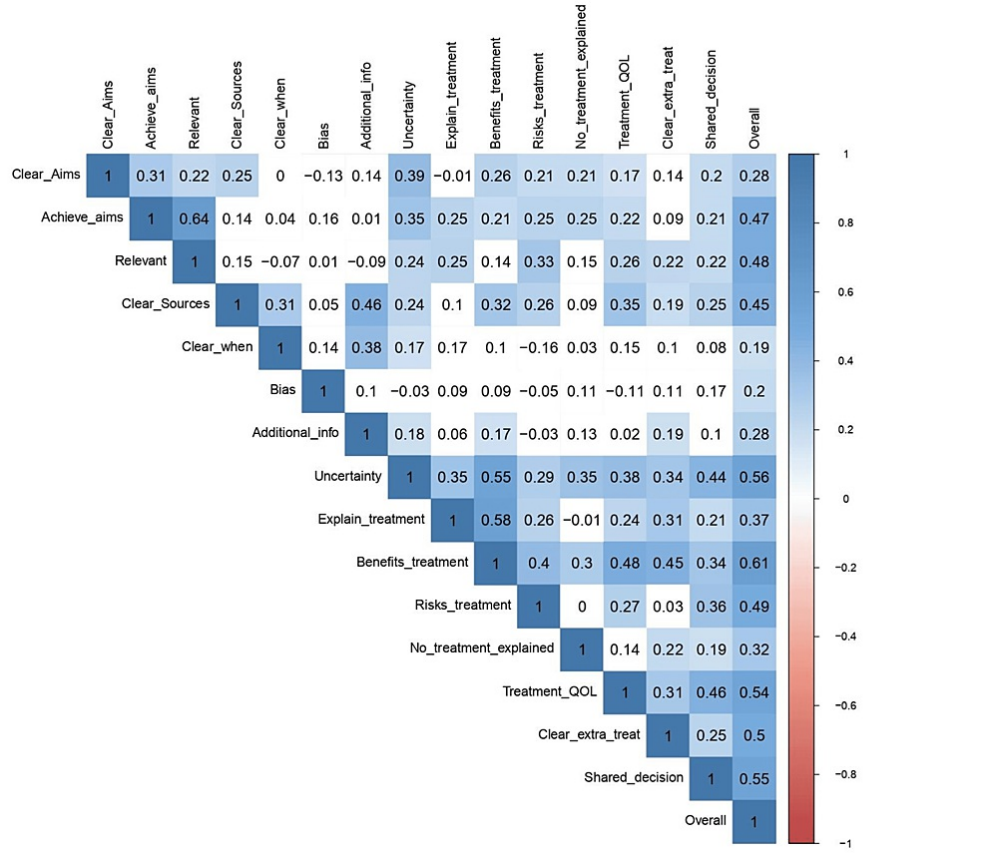
The correlation of views, likes, and overall quality for each of the questions.

Using Table 5 to show which key phrase in Figure 4 correlates with each question from the survey, The correlation between the average rating, likes, and views was assessed in a graph shown in Figure 4. The correlation between likes and views was 0.22. The correlation between likes and the overall quality of the video was -0.068. The correlation between overall quality and views was 0.03, as shown in Table 6.

**Table 5:** Shows which abbreviations from Figure 4 represent each question.

Abbreviations	Questions they correlate to
Clear aims	Does the video have clear aims? (Does it talk about the purpose of the video, who it is targetting, what it is about)
Achieve aims	Does the video achieve its aims? (5= has all the info you would expect, 1= completely misses the mark, and 2-4 somewhere in the middle)
Relevant	Is the video relevant? Does it address all the questions that someone would ask? Are recommendations and suggestions appropriate?
Clear sources	Is it clear what sources of information were used to compile the video? (MD/DO talking with no reference = 3, Non MD/DO talking with no reference should be 1)
Clear when	Is it clear when the information reported was produced?
Bias	Is there any bias in this video? (look for terms that display emotions, and paint over positive/ negative images of a topic)
Additional info	Does it provide an additional source of information?
Uncertainty	Does it refer to any areas of uncertainty? Look for statements that generalize treatment for everyone, 2-4= they mention these, but do not go into details)
Explain treatment	Does it explain how treatment works?
Benefit treatment	Does it describe the benefits of treatment? (If they say "it will prevent/fix CAD" without any detail, rate a 1)
Risk treatment	Does it describe the risks of treatments? (2-4: some but not all treatment risks are discussed)
No treatment explained	Does this video describe what happens when no treatment is used? (Look for any risks or benefits of postponing treatment, monitoring the condition before undergoing treatment, or permanently forgoing treatment)
Treatment QOL	Does it describe how treatment choices could affect the overall quality of life? (effects on day-to-day activity, effects on family, caregivers, friends) (blank statements like "it will impact function" will be a 3)
Clear extra treat	Is it clear that there is more than one option to treat/ manage CAD? (just mentioning an additional option is a 5, no additional description is necessary)
Shared decision making	Does the video mention any shared decision-making? (i.e., Discussions with family/ friends, "Talk to your doctor and see what options are best for you?")
Overall	Rate the overall quality of the video on one to five

**Table 6:** The overall correlations when likes, views, and overall quality of videos were compared to each other.

Comparison	Correlations
Likes-view	0.22
Likes-overall	-0.07
Views-overall	0.03

## Discussion

Clinical implications

YouTube is one of the most popular websites in the United States, especially when it comes to providing medical information to patients. However, according to previous studies, YouTube videos have had a poor track record of providing patients with good-quality information about various medical conditions. This study continued that trend, with a poor average overall quality rating across all search categories. One of the biggest areas of improvement has to come from discussions about shared decision-making, quality of life after the procedure, and the risks of treatment. Discussing these topics in the videos and promoting continued discussion with their provider will give them a better sense of what to consider when deciding on a treatment, especially a procedure. For instance, a discussion about the usual recovery time for patients undergoing a cardiac catheterization in a YouTube video might alleviate anxiety about a long hospital stay and encourage further discussion with their medical provider. 

Another area of improvement based on the survey results was the provision of a clear source of information and additional resources to viewers who wanted to learn more. When providing medical information, especially recent changes to medication, it is important to support your claims with research articles so the viewer can form their own opinion. It has been shown that during the COVID-19 pandemic, preference in mainstream news sources had a strong effect on how much effort their viewers placed on infection prevention [22]. Efforts must be made to ensure the reliability of information as well as openness to discussion about said information. 

From this study, it was also found that videos with board-certified physicians had a significantly higher average than those without a board-certified physician. This further emphasizes the importance and necessity for physicians to take a larger role in making patient education content on social media or video platforms such as YouTube. We should further encourage YouTube personalities who happen to be physicians to provide medical information that is both expansive and engaging to the viewer.

The final important takeaway from this study is that what viewers saw and liked poorly correlates to the overall quality of the video. This shows that there is a disconnect between how valuable the information was to the patient and what videos were viewed and liked. One way to improve this disconnect is to create more content with the patient in mind. Several videos at the top of the searches were created by either medical school test preparation companies or private companies. From this, it would be reasonable to assume that the target audience for these videos would be medical students preparing for board reviews or private companies trying to advertise a product. The patient's quality of life and risk of a certain procedure-information a patient would like further details about-may be brushed over in the board review videos to prevent distractions from highly testable content.

Future directions

After this study, the next step would be to create a video that talks about all the information in the DISCERN criteria and is entertaining for the viewer. This video could be compared to a "3.0"-rated video to see if there is a significant change in results. This would be a good audit to see if making these high-quality videos could make steady improvements in the outpatient clinic. In the studies above, there has been mention of how visual aids help educate patients on mechanical thrombectomy. It would be interesting to see if similar success can be achieved in the preventative stages of coronary artery disease. 

Additionally, long-term longitudinal studies can be done to assess the impact that medical education resources have on patient behavior. Following long-term trends and patterns in patient responses to these resources can help us tailor resources to the needs of patients, potentially increasing compliance. These studies can be extended beyond YouTube and include other platforms like Instagram and Facebook (Meta Platforms, Inc., Menlo Park, California, United States). Chat GPT (OpenAI, San Francisco, California, United States) and Google Gemini (Google LLC, Mountain View, California, United States) would also be interesting topics of study, given the recent rise of AI as a platform for information. Finally, comparative analyses can be performed to analyze the difference between high-quality YouTube videos and standard content, which can lead to a further understanding of how content can impact content quality and decision-making. 

Limitations

In this study, some important limitations need to be assessed. When assessing the quality difference between videos with cardiology-related physicians and non-cardiology-related physicians, there was too little of a sample study to make a reasonable conclusion. The length cut-off for videos may have eliminated some more expansive videos that discussed all the details necessary to adequately educate the patient. Eliminating videos that are based on time and language also limits the diversity and sample size of the videos. Also, YouTube is an ever-changing video platform, so the order of videos with the most views may have changed since the study began. 

Although most studies only included one or two viewers, we limited the number of viewers to three, introducing subjectivity and limited generalizability. Although all three of the viewers have a medical background, it is important.

Despite DISCERN criteria being considered one of the gold standards for assessing the reliability of medical information, focusing only on DISCERN criteria may ignore important aspects of video quality and patient comprehension. Additionally, DISCERN criteria were originally used to assess medical text on websites as opposed to YouTube videos. However, due to the lack of standardized methods of analyzing YouTube videos, this scoring system was employed.

## Conclusions

YouTube is a great source for providing medical education to people of all socioeconomic statuses. However, the quality of videos, especially those on coronary artery disease, must improve in promoting shared decision-making and providing details about the quality of life and possible risks of a procedure. Therefore, physicians should make more of an effort to educate patients through this platform. Finally, there is a disconnect between the viewers the popular videos target as well as the needs of the patient.
